# Acute effect of high-intensity interval training versus moderate-intensity continuous training on appetite-regulating gut hormones in healthy adults: A systematic review and meta-analysis

**DOI:** 10.1016/j.heliyon.2023.e13129

**Published:** 2023-01-21

**Authors:** Mingzhu Hu, Zhaowei Kong, Qingde Shi, Jinlei Nie

**Affiliations:** a University of Macau, Macao, China; b Macao Polytechnic University, Macao, China

**Keywords:** Sprint interval training, Appetite-regulating hormones, Ghrelin, Glucagon-like peptide-1, Peptide YY

## Abstract

**Background:**

Exercise intensity has been suggested to influence acute appetite-regulating gut hormone responses after exercise. High intensity interval training (HIIT) with near maximal to maximal intensity or sprint interval training (SIT) with supramaximal intensity might induce greater effects on gut hormones compared to moderate intensity continuous training (MICT), while current findings were inconsistent regarding the effects of these popular training methods.

**Objective:**

This systematic review and meta-analysis aimed to synthesis the findings in the literature and explore the impact of exercise modality on acylated ghrelin (AG), glucagon-like peptide-1 (GLP-1) and peptide YY (PYY).

**Methods:**

After searching the major databases (PubMed, Web of science and ScienceDirect, Scopus, Cochrane Library) to find articles published up to May 2022, twelve studies that compared hormone responses to HIIT/SIT and MICT were identified and included in the analysis.

**Results:**

A random-effects meta-analysis showed that HIIT/SIT and MICT decreased AG concentration and increased GLP-1 and PYY concentration compared with no exercise control group, while interval training protocols, especially SIT protocols, elicited greater effect sizes in suppressing AG levels at all of the analysed time points and PYY immediately post-exercise compared to MICT.

**Conclusion:**

Acute SIT with lower exercise volume appears to be a more advantageous approach to decrease plasma AG concentration and potentially suppress hunger to a greater extent compared to MICT, despite the similar effects of HIIT/SIT compared to MICT in increasing anorectic hormones (i.e., GLP-1 and PYY). Future studies are needed to further investigate the impact of moderators (e.g., gender, body composition and exercise mode) on the variability of changes in gut hormones after interval trainings.

## 1 Introduction

How exercise affects appetite-related gut hormones that regulate food intake [1], as one of the mechanisms explaining the effectiveness of exercise for weight loss, has been a subject of discussion in the literature over the years [[2], [3], [4]]. Together with signals from adiposity (e.g. leptin) and the pancreas (e.g. insulin), gut hormones released from the gastrointestinal tract act as peripheral signals to the receptors in the hypothalamic arcuate nucleus to regulate nutritional status and achieve energy homeostasis by suppressing or stimulating appetite [5]. These hormones, therefore, are categorised into anorectic hormones that suppress feelings of hunger (i.e., peptide YY (PYY) and glucagon-like peptide 1 (GLP-1)) in response to food intake [6,7] and orexigenic hormones which stimulate appetite (i.e., ghrelin) [8,9]. It has been suggested that changes in the concentration of bioactive forms of these hormones (i.e., acylated ghrelin (AG), PYY_3-36,_ GLP_7-36_) were more relevant and distinguishable when analysing exercise-induced effects on appetite [10,11]. Specifically, AG is responsible for the endocrine and pituitary activities rather than nonacylated ghrelin despite its small proportions of the total ghrelin compared to the nonacylated form (80–90%) [12], while PYY_3-36_, converted from PYY_1-36_, is the major circulating form of the peptide YY in the blood and is more selective for the Y_2_ receptor, producing anorectic effects [6]. Previous meta-analyses have demonstrated small to moderate effects of acute exercises on suppressing AG and increasing total PYY and GLP-1 concentrations in lean or overweight and obese participants [13,14]. Exercise parameters, especially exercise intensity, were proposed to exert an influence on the magnitude of the impact of exercise on concentrations of gastrointestinal hormones, as greater reductions in AG were more frequently observed in exercise with higher intensity [15]. Although exercise volume may also affect hormone releases, very low volume high-intensity exercise was reported to induce significant changes in appetite-related hormones and suppress appetite as well [16].

Moderate intensity continuous training (MICT) has been traditionally recommended for managing body composition and improving cardiovascular fitness, while high-intensity interval training (HIIT) consisting of repeated short high-intensity bouts interspersed with recovery has become a popular option to achieve similar health goals in a more efficient manner compared to MICT [[17], [18], [19]]. As a form of HIIT with sprinting intervals at the highest end of the intensity spectrum, sprint interval training (SIT) with a lower volume has received increased attention in recent years [20]. Given that exercise-induced responses in appetite-regulating hormones could be intensity-dependent, HIIT/SIT with near-maximal to supramaximal intensity might be a more desirable strategy compared with MICT to suppress orexigenic hormones and increase anorectic hormones, thus triggering exercise-induced anorexia. Accumulating evidence has shown the greater inhibition of AG secretion and increased concentrations of PYY and GLP-1 after HIIT/SIT compared to MICT [[21], [22], [23], [24]]. However, several studies failed to observe significant effects of HIIT/SIT compared to MICT or suggested that both HIIT/SIT and MICT could not induce changes in anorectic hormones (i.e., PYY and GLP-1) compared to the non-exercise control group [[25], [26], [27], [28]]. Given that the findings from these individual studies with relatively small sample sizes and varied methodology yielded no consistent conclusions on whether HIIT/SIT could lead to greater or comparable effects on appetite-related gut hormones compared to MICT and no systematic review and meta-analysis has been conducted so far, there is a need to quantify the effect with a pooled estimate and provide comprehensive information to clarify the effects of HIIT or SIT compared to MICT on appetite-related hormones.

Therefore, the current study aimed to synthesise the evidence from available studies that compared the effects of acute sessions of HIIT/SIT and MICT on three major appetite-related hormones including AG, PYY and GLP-1, presented as total plasma concentrations and active forms in healthy individuals. The effects of HIIT/SIT and MICT compared to the control group without training intervention would also be analysed to confirm the impact of these two different training modalities on these hormones. Although the area under the curve (AUC) reporting hormone concentrations by time was used as the only metric in previous reviews [13,14], analyses only based on AUC values might underestimate the effect of exercise given the confounding AUC estimations in the included studies (e.g., variations in the timing for exercise, resting duration and sampling rate). Therefore, to reveal the effect of exercise on gut hormones and capture the change in gut hormones at different time-points, analyses in the present study would be performed for concentrations of the targeted hormones quantified as the AUC and the hormone concentrations immediately post-exercise and 30- to 90-min post-exercise. We hypothesised that both HIIT/SIT and MICT would exert effects on appetite-related hormones and HIIT/SIT would result in greater effects than MICT in plasma concentrations of AG, PYY and GLP-1.

## 2 Methods

The current review was conducted in accordance with the PRISMA guideline (Preferred Reporting in Systematic Reviews and Meta-Analyses) [29] and was registered in the PROSPERO database (registration number: CRD42022337521).

### 2.1 Search strategy

The literature search was performed using major databases including PubMed, Web of Science and ScienceDirect, Scopus and Cochrane Library to find records that have undergone peer-review and were published in English until May 1, 2022. The search strategy combined the following two groups of key words (i.e., exercise intervention and outcome) applied to “all fields”, “title” and “abstract”: “exercise”, OR “physical activity”, OR “high intensity training” AND “appetite”, OR “appetite hormone”, OR “gastrointestinal hormones”, OR “appetite-regulating hormones”, OR “ghrelin”, OR “acylated ghrelin”, OR “PYY”, OR “peptide YY”, OR “PYY_3-36_”, OR “glucagon-like peptide-1”, OR “active GLP-1”, OR “GLP-1(7–36)”, OR “GLP-1(9–36)”. The detailed search strategy and examples of full syntaxes for each database are listed in supplementary Table S1.

### 2.2 Eligibility criteria

The screened articles would be included in the current review if they met the following criteria: 1) participants in the study were healthy adults with no habits of smoking, no chronic diseases and who were not taking medication, with no restrictions on participants’ weight or physical fitness levels; 2) the study involved both HIIT and MICT as exercise groups and included a control group which was identical to that of the training condition(s) but without exercising; 3) the study measured appetite regulating hormones including ghrelin and/or PYY and/or GLP-1 expressed by plasma total volume or the active form (i.e., AG, PYY_3-36,_ active GLP-1) before and after the experimental sessions. The outcomes could be presented with data collected at separate time points (e.g., 60-min post-exercise) and/or the area under curve values (AUCs) during the experimental session. HIIT was classified as exercises with intervals performed at near maximal, maximal or supramaximal intensity (i.e., sprinting) (≥85% of the maximum heart rate (HR_max_) or ≥80% VO_2peak_) [30]. HIIT protocols with all-out efforts were termed SIT and were included in the review. MICT that acted as the comparator to HIIT was defined as continuous training at moderate intensity (55–70% HR_max_ or 40–60 VO_2peak_) [31]. There were no restrictions regarding the modality or duration or the volume of the training protocol.

Studies were excluded if there were other treatment on top that might affect appetite or appetite-regulated hormones (e.g., dietary modifications) and/or if the trial sequence or allocations of the participants did not go through a randomisation process. The reason we excluded the subjects with diseases or non-adult participants is that these subjects could have contraindications to exercise, especially when performing HIIT or SIT with all-out effort. Moreover, the medications that subjects with diseases consumed could influence physiological responses to exercise training protocol.

### 2.3 Data extraction and synthesis

Data were extracted from the included studies and entered into a spreadsheet as follows: the first author of the study and the published year, participants’ characteristics (age, gender, body mass index (BMI), fitness level: inactive or active), descriptions of the HIIT and MICT intervention (mode intensity, duration of the training), outcomes for appetite-regulating hormone concentrations (pg•mL^−1^) (means and standard deviations in each group, statistical differences between exercises protocols including HIIT and MICT and the control group and statistical differences between HIIT and MICT). Standard error of the mean (SEM) was converted to standard deviations and pmol•L^−1^ values of the hormone concentration were converted to pg•mL^−1^ as follows: multiplied by 4 for PYY, 3.38 for acylated ghrelin and 3.297 for GLP-1 [14]. Data from figures were extracted using an online tool (WebPlotDigitizer; https://automeris.io/WebPlotDigitizer) and the authors were contacted for raw data if the required data were not directly available in the article.

### 2.4 Risk of bias

Risk of bias of the included studies was assessed using The Cochrane Risk of Bias tool [32], which consists of three major domains: selection bias in the randomisation process, attrition bias due to missing outcome data and reporting bias due to selective reporting. Risk of bias for blinding could not be assessed, since the participants or the personnel would inevitably know details of the exercise interventions. Risk of bias arising from period and carryover effects in crossover trials were assessed using the Revised Cochrane risk-of-bias tool for randomised crossover trials.

### 2.5 Analysis

All meta-analyses were performed using Comprehensive Meta-Analysis software (version 3, New Jersey, USA). The random effects model was used to analyse the standardised mean difference (SMD) in acute effects of exercise (i.e., HIIT/SIT and MICT) versus the control group (CON) and HIIT/SIT versus MICT on appetite regulating hormones including plasma concentrations of AG, total PYY, PYY_3-36_ and total GLP-1 at different time points (i.e., immediately post-exercise and 30- to 90-min post-exercise) and presented as AUC values. Given that all studies included in the meta-analysis were cross-over trials and provided a wash-over period to avoid carry-over effects, sensitivity analyses were performed using standard paired differences and correlation coefficients of 0.6, 0.7, 0.8 and 0.9. Because no significant differences in the results were found after performing the sensitivity analysis, a default correlation coefficient of 0.5 was used to conduct the meta-analysis. Data from studies that included both HIIT and SIT protocols or utilised two categories of participants (i.e., men vs. women) were separated as study arms with halved sample sizes in each arm to reduce the likelihood of overweighting. Meta-analyses for the effects of HIIT compared to MICT on total ghrelin and active GLP-1 in were not performed due to limited data from the included studies (i.e., <3 studies that provided data). Data for SIT was combined with data for HIIT to compare with MICT because SIT is considered a type of HIIT with the maximal intensity. Nevertheless, outcomes from the meta-analysis were grouped by protocol types (i.e., SIT or HIIT) when more than one study was included in each subgroup to explore possible different effects of SIT and HIIT in relative to MICT. Effect size (SMD) are interpreted as trivial if < 0.2, as small if 0.2–0.3, as moderate if 0.5 and as large if > 0.8 [33]. Sensitivity analyses were performed by leaving out one study at a time to examine whether the results were largely impacted by any over-weighted study. Visual inspection of funnel plot and Egger's regression test were performed to assess publication bias for analyses with more than 10 study arms. I-squared statistic (I^2^) was used to identify levels of heterogeneity (low heterogeneity: I^2^ <25%, moderate heterogeneity: I^2^ = 25–75% and high heterogeneity: >75%) [34]. Restricted maximum likelihood random-effects meta-regression using BMI as the moderator or subgroup analysis using gender and fitness level (active or inactive) of the participants, measurement time (≤1 h or>1 h) as moderators would be used to explore potential reasons for moderate to high heterogeneity levels (I^2^>50%) in the meta-analyses with more than 10 study arms that compared HIIT/SIT with MICT.

## 3 Results

A total of 12 studies [16,[21], [22], [23], [25], [26], [27], [28],^35^,[24], [36], [37]] were included in the current review after systematic searching and screening. The procedure for study selection and reasons for exclusion are shown in Fig. 1.

**Fig. 1:**
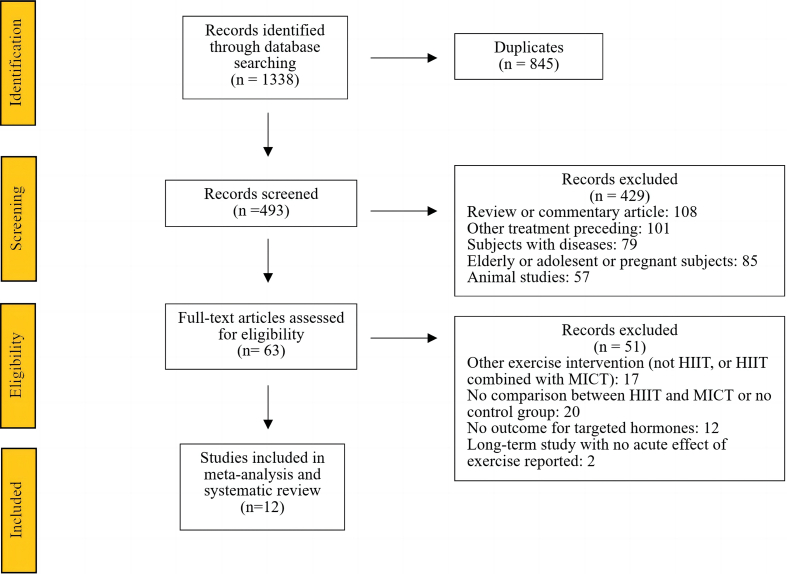
Flow diagram of screening and selection of articles for review.

### 3.1 Study characteristics

Risk of bias evaluations of the included studies are presented in supplementary Figure S1. All of the included studies had low bias from the randomisation process, while it was unclear about the selection bias arising from the allocation process, as all studies except one reported allocation concealment [28]. More than half of the studies (n = 7) had low levels of attrition bias [16,[21], [23],^26^,^27^,[36], [37]], while the other five studies resulted in unclear levels of attrition bias given that no sufficient information was provided [22,25,28,[24], [35]]. Levels of reporting bias were low in five of the included studies [16,21,27,[24], [36]], while it is unclear about the other seven studies [[22], [23], [25], [26],^35^,[37], [38]]. Evaluations of the carry-over effect demonstrated low levels of carry-over effect in the majority of the studies (n = 11) since these studies provided one to two weeks of wash-over periods between the experimental session; one study did not provide information regarding the wash-over periods [16].

Study characteristics and outcomes of studies are summarised in Table 1 [16,[21], [22], [23], [25], [26], [27],^35^,^36^,^37^]. A total of 158 participants were involved in the 12 included studies, while men account for the majority of participants (i.e., 116 males, 73%). Four studies involved overweight to obese individuals (i.e., BMI>27 kg•m^−2^) [23,27,28,35], while the other eight studies recruited normal weight individuals with a mean BMI ranging from 23 to 24.8 kg•m^−2^. Regarding the fitness status, participants from the four studies involving overweight to obese individuals were also inactive. Five out of the eight studies involving normal weight individuals reported that the participants were also active, while the other three studies [16,[21], [24]] did not provide exact information on the fitness status of their participants. Five of the 12 studies [23,28,35,[24], [36]] analysed HIIT protocols with 60- or 240-s bouts at 85–100% VO_2peak_, while most studies (n = 9, including two studies analysed both HIIT and SIT) investigated SIT protocols with 8- to 30-s sprinting. Cycling was adopted as the exercise mode in the majority of the included studies (n = 10); only two of the 12 studies utilised running [26,28]. Nearly half of the included studies (n = 5) matched work done in HIIT and/or SIT with MICT [23,27,35,[24], [36]], while the other half either used HIIT/SIT with lower work output than MICT (n = 5) [16,21,22,[26], [37]] or did not report the total work done by the exercise groups (n = 2) [25,28].

**Table 1:** Study characteristics and outcomes of the included studies.

Study	Participants			Exercise training intervention		Outcomes			*Measurement time (AUC duration)*
	N(gender)	BMI (kg•m^−2^)	Fitness level	HIIT/SIT	MICT	Ghrelin	GLP-1	PYY	
Deighton et al. (2013) [24]	12(M)	23.7 ± 3.0	NR	HIIT: 10 × 240 s cycling at 85–90% VO_2peak_ +120 s rest	cycling at 60% VO_2peak_ for 60-min *(work done matched with HIIT)*	NM	NM	PYY_3-36_ ↑	baseline, 2 h–pre-exercise, 3 h–immediately post exercise, 3.75, 5, 6, 7, 8 h (3.75 h)
								Favored MICT immediately end exercise	
								Favored HIIT in hours after completion	
Deighton et al. (2013) [21]^*a*^	12(M)	24.2 ± 2.9	NR	SIT: 6 × 30 s sprinting against 7.5% of body mass+ 240 s passive recovery	cycling at 68.1 ± 4.3% VO_2peak_ for 60-min	Acylated Ghrelin↓	NM	Total PYY↑	baseline, 2 h–pre-exercise, 3 h–immediately post exercise, 3.5, 4.5, 5.5, 7 and 8 h
						Favored SIT		Favored MICT	
Hallworth et al. (2017)[25]	9(F)	23.5 ± 2.8	active	SIT: 6 × 30 s sprinting against 10% of body mass + 240 s passive recovery	cycling at 65% VO_2peak_ for 30-min	NM	GLP-1↑	Total PYY→	pre-exercise, ∼0.67 h–immediately post exercise, and 2 h
							Similar		
Hallzell et al. (2017) [22]	10 (M)	23.7 ± 2.2	active	SIT: 6 × 30 s sprinting against 10% of body mass + 240 s passive recovery	MICT: cycling at 65% VO_2peak_ for 30-min	NM	GLP-1 →	Total PYY↑	pre-exercise, ∼0.67 h–immediately post exercise, and ∼2.17 h (∼2.17 h)
					HICT: cycling at 85% VO_2peak_ for 30-min			Favored SIT (immediately post exercise)	
Hallzell et al. (2017) [37]^a^	21 (11 F, 10 M)	23.7 ± 2.2	active	SIT: 6 × 30 s sprinting against 10% of body mass + 240 s passive recovery	cycling at 65% VO_2peak_ for 30-min	NM	GLP-1↑	Total PYY↑	pre-exercise, ∼0.67 h –immediately post exercise, and ∼2.17 h
							Similar (effect of sex: greater in females)	Favored SIT (immediately post exercise) (effect of sex: greater in males)	
Islam et al. (2017) [26]	8 (M)	24.8 ± 2.3	active	SIT: 4 × 30 s ‘all-out’ running + 4-min rest	MICT: running at 65% VO_2max_ for 30-min	Acylated Ghrelin ↓	Active GLP-1↑	Total PYY↑	pre-exercise, ∼0.67–immediately post exercise, 1.17, 2.17 h (∼2.17 h)
							AUC↑		
					HICT: running at 85% VO_2max_ for 30-min	Favored SIT	Favored MICT (immediately post exercise)	Similar	
							Favored SIT for AUC and 30-min post exercise		
Larsen et al. (2018) [35]	11 (M)	28 ± 3	inactive	HIIT: 60 s cycling at 100% VO_2peak_ +240 s active recovery at 50% VO_2peak_ for 30-min	cycling at 60% VO_2peak_ for 30-min *(work done matched with SIT)*	Acylated Ghrelin ↓	NM	Total PYY→	pre-exercise, 0.5 h–immediately post exercise, and the next morning after the exercise
						Favored HIIT (30-min post exercise)			
Martins et al. (2014) [27]	12 (7 F, 5 M)	32.3 ± 2.7	inactive	SIT: 8 s sprinting at 85–90% HR_max_+ 12 s active recovery for 18 ± 3 min	cycling at 70% HR_max_ for 27 ± 6 min *(work done matched with SIT)*	Acylated Ghrelin ↓	GLP-1↑	PYY_3-36_ →	baseline, 0.5, 1, ∼1.33 h–pre - exercise, 1.67 h–immediately post exercise, 2, 2.5, and 3 h (3 h)
				SIT (half volume): same protocol as above for 9 ± 2 min		Similar	Similar		
Matos et al. (2018) [28]	12 (M)	35.5 ± 4.5	inactive	HIIT: 10 × 60 s running at 90% of maximal heart rate with 60 s recovery	running at 70% of maximal heart rate for 20 min	NM	GLP-1↑in MICT	NM	pre-exercise, ∼ 0.42 h–immediately post exercise, and 1.42 h
							Favored MICT (post 1 h)		
Metcalfe et al. (2015) [16]	8 M *(Study 2*)	25 ± 4	NR	SIT (REHIT): 2 × 20 s sprinting + 10 min cycling at 60 W	cycling at 50% of VO_2max_. for 30 min	Acylated Ghrelin ↓	NM	Total PYY→	pre-exercise, ∼ 0.5 h–immediately post exercise, 0.75, 1 and 2 h (2 h)
						Favored SIT			
Panissa et al. (2016) [36]	20 (9 F,11 M)	∼23 (F)	active	SIT: 60 × 8 s sprinting + 12 s passive recovery for 20-min (8 min of effort and 12 min of pause)	cycling at 60% of 100% of maximal load attained in incremental test for 19 ± 2 min *(work done matched with SIT and HIIT)*	Acylated Ghrelin ↓ (AUC) only in SIT	NM	PYY_3-36_ → (effect of sex: greater in males)	baseline, 2 h–pre-exercise, 2.5 h–immediately post exercise, 3.25, and 4 h
		∼24.6 (M)		HIIT: 60 s cycling at 100% of maximal load attained in incremental test + 60 s passive recovery for 17 ± 2 min (9 min of effort and 8 min of pause)					
Sim et al. (2014) [23]	17 (M)	27.7 ± 1.6	inactive	SIT: 15 s sprinting at 170% VO_2peak_ + 60 s active recovery for 30-min	cycling at 60% VO_2peak_ for 30-min *(work done matched with SIT)*	Acylated Ghrelin ↓	NM	Total PYY→	pre-exercise, 0.5 h–immediately post exercise, 1 and 1.5 h
				HIIT: 60 s cycling at 100% VO_2peak+_240 s active recovery for 30-min		Favored SIT			

a = the latter published study that published in the same year by the same first author; F = Female; M = male; HR_max_ = Maximum heart rate; $\dot{\text{V}}$ O_2max_ OR $\dot{\text{V}}$ O_2peak_ = peak oxygen uptake; ∼ = rough value calculated by the provided data from the included study; NR = not reported; PYY = Peptide YY; GLP-1 = Glucagon-like peptide 1; ↑OR ↓ = significantly higher OR lower levels after exercise session (s) compared with resting control trial; → = no significant difference induced by exercise compared to resting control trial; NM = not measured; AUC = the area under curve; h = hour.

### 3.2 Main outcomes

For each appetite-regulating hormone (acylated ghrelin, total ghrelin, total PYY, PYY_3-36_), two major analyses were performed using available data: 1) comparisons between exercise groups (EXE: HIIT, SIT and MICT) and the control group (CON) with no exercise intervention; and 2) comparisons between HIIT/SIT and MICT. Outcomes of the meta-analyses (ES: effect size, CI: confidence interval, p-value, number of study arms, I^2^) are summarised in supplementary Table S2. All figures of funnel plots for analyses with more than 10 study arms were presented in the supplementary file (Figure S6 -16).

#### 3.2.1 Acylated ghrelin

Seven studies [16,21,23,26,27,35,36] reported changes in AG concentration after HIIT/SIT and MICT. All seven of the studies reported significant suppression of AG concentration after HIIT/SIT and MICT compared to the non-exercise control group and greater effects of HIIT/SIT compared to MICT were observed in six of the seven studies involving both inactive overweight to obese individuals (three studies) and active and/or normal weight individuals in both sexes (67 males and 16 females). Four studies used energy matched HIIT/SIT and MICT protocols and three out of the four studies favored HIIT/SIT on the suppression of AG compared to MICT [23,35,36]. One of the four studies did not find any significant differences between MICT and SIT in terms of suppressing AG concentrations [27].

Outcomes of meta-analysis of the acute effect of EXE compared to CON on AG concentrations are presented in supplementary Figure S2. Compared to the CON, there were significant effects of exercise on decreasing acylated ghrelin concentration immediately post-exercise (ES = −1.078 [−1.427, −0.729], *p* < 0.001, study arms = 17, I^2^ = 54.8%), 30- to 90-min post-exercise (ES = −0.387 [−0.747, −0.026], *p* = 0.035, study arms = 17, I^2^ = 61.8%) and in AUC values (ES = −0.705 [−1.090, −0.320], *p* < 0.001 study arms = 8, I^2^ = 33.4%). The effect size was increasingly smaller from immediately post-exercise (large effects) to AUC values (large and moderate effects) to 30- to 90-min post-exercise (small to moderate effects). Sensitivity analyses with one study removed did not significantly alter the overall effects. Moderate to high heterogeneity levels were found in analyses of AG immediately post-exercise (I^2=^54.8%), 30- to 90-min post-exercise (I^2^ = 61.8%) and AUC (I^2^ = 33.4%). Visual inspection of the funnel plot and Egger's regression tests suggested potential publication bias in the analysis of AG immediately post-exercise (Egger's regression intercept = − 7.220, confidence interval: −10.78 to −3.65, *p* < 0.001). Although the funnel plot of the analysis for 30–90 min post-exercise shows evidence of publication bias, Egger's regression test was not significant (*p* = 0.13). Subgroup and meta-regression analyses were performed to investigate potential moderator variables that contributed to the variation of the true effect; the results are presented in Table 2. The results showed that the effect sizes were related to the protocol type (i.e., HIIT, SIT or MICT) (*p* = 0.001) and gender (*p* < 0.001) in the analysis of AG immediately post-exercise, while BMI, fitness level and protocol types (*p* < 0.05) might have influenced the effect size in the analysis of AG 30- to 90-min post-exercise.

**Table 2:** Summary of moderator variable analysis for EXE (HIIT/SIT and MICT) vs. CON by sub-group and meta-regression.

Acylated ghrelin-immediately post (study arms: 17)							
Moderator	Subgroup	Coefficient	95% Lower	95% Upper	Z-value	2-sided P-value	Set
**BMI**	(study arms: 17)	0.149	−0.051	0.348	1.46	0.145	
**Fitness level** (Reference group: NR)	Active (study arms: 8)	−0.637	−1.281	0.006	−1.94	0.052	Q = 5.57, df = 2, p = 0.062
	Inactive (study arms: 5)	−1.263	−2.586	0.061	−1.87	0.061	
**Protocol types** (Reference group: SIT)	HIIT (study arms: 3)	0.064	−0.697	0.826	0.17	0.868	Q = 13.95, df = 2, p = 0.001*
	MICT (study arms: 7)	0.908	0.400	1.416	3.5	0.0005*	
**Gender:** (Reference group: Female)	Male (study arms: 14)	−1.359	−2.142	−0.576	−3.4	0.001*	
**Acylated ghrelin 30**–**90 min post** (study arms: 17)							
**BMI**	(study arms: 17)	0.206	0.015	0.397	2.12	0.034*	
**Fitness level** (Reference group: NR)	Active (study arms: 8)	0.667	0.06	1.275	2.15	0.031*	Q = 16.27, df = 2, p = 0.0001*
	Inactive (study arms: 5)	−1.604	−2.857	−0.35	−2.51	0.012*	
**Protocol types** (Reference group: SIT)	HIIT (study arms: 3)	0.894	0.203	1.586	2.54	0.011*	Q = 10.46, df = 2, p = 0.005*
	MICT (study arms: 7)	0.692	0.206	1.177	2.79	0.005*	
**Gender:** (Reference group: Female)	Male (study arms: 14)	−0.246	−0.991	0.498	−0.65	0.516	
**GLP-1-immediately post** (study arms: 12)							
**BMI**	(study arms: 12)	−0.216	−0.537	0.105	−1.32	0.186	
**Fitness level** (Reference group: Inactive)	Active (study arms: 8)	−1.842	−4.978	1.295	−1.15	0.25	
**Protocol types** (Reference group: SIT)	HIIT (study arms: 1)	0.647	−0.623	1.917	1	0.318	Q = 1.38, df = 2, p = 0.501
	MICT (study arms: 6)	0.287	−0.275	0.85	1	0.317	
**Gender:** (Reference group: Female)	Male (study arms: 8)	−1.143	−1.794	−0.492	−3.44	0.001*	
**Total PYY immediately post** (study arms: 17)							
**BMI**	(study arms: 17)	−0.253	−1.04	0.533	−0.63	0.528	
**Fitness level** (Reference group: NR)	Active (study arms: 10)	1.361	0.51	2.211	3.13	0.002*	Q = 16.31, df = 2, p < 0.001*
	Inactive (study arms: 3)	1.555	−1.084	4.195	1.15	0.248	
**Protocol types** (Reference group: SIT)	HIIT (study arms: 1)	−0.408	−1.892	1.076	−0.54	0.59	Q = 4.22, df = 2, p = 0.121
	MICT (study arms: 8)	−0.593	−1.16	−0.026	−2.05	0.04*	
**Gender:** (Reference group: Female)	Male (study arms: 13)	0.809	0.004	1.615	1.97	0.049*	
**PYY**_**3-36**_**immediately post** (study arms: 10)							
**BMI**	(study arms: 10)	−0.041	−0.233	0.15	−0.42	0.672	
**Fitness level** (Reference group: NR)	Inactive (study arms: 8)	2.404	0.875	3.933	3.08	0.002*	
**Protocol types** (Reference group: SIT)	HIIT (study arms: 3)	0.438	−0.981	1.857	0.61	0.545	Q = 0.37, df = 2, p = 0.832
	MICT (study arms: 4)	0.196	−1.041	1.433	0.31	0.756	
**Gender:** (Reference group: Female)	Male (study arms: 7)	1.396	−0.059	2.851	1.88	0.06	
**PYY**_**3-36**_**30–90 min post** (study arms: 10)							
**BMI**	(study arms: 10)	−0.116	−0.322	0.09	−1.1	0.271	
**Fitness level** (Reference group: NR)	Inactive (study arms: 8)	0.967	−0.675	2.608	1.15	0.248	
**Protocol types** (Reference group: SIT)	HIIT (study arms: 3)	−0.439	−1.973	1.095	−0.56	0.575	Q = 0.52, df = 2, p = 0.771
	MICT (study arms: 4)	−0.481	−1.835	0.873	−0.7	0.486	
**Gender:** (Reference group: Female)	Male (study arms: 7)	2.119	0.546	3.691	2.64	0.008*	

NR = Not reported; * = significant effect of the moderator.

When compared with MICT (Fig. 2), HIIT/SIT protocols demonstrated greater effects on reducing concentrations of AG immediately post-exercise (ES = −0.729 [−1.040, −0.418], *p* < 0.001, study arms = 10, I^2=^0%), 30- to 90-min post-exercise (ES = −0.380 [−0.659, −0.100], *p* = 0.008, study arms = 11, I^2^ = 18.3%) and in AUC values (ES = −0.696 [−1.345, −0.047], *p* = 0.036, study arms = 4, I^2^ = 52.5%). The effect sizes were moderate to large immediately post-exercise and in AUC values but was small to moderate at 30- to 90-min post-exercise. The one-study-removed procedure did not impact the overall effect. Subgroup analysis showed that SIT generated significantly greater effects on reductions of AG concentration 30- to 90-min post-exercise than MICT, while differences between HIIT and MICT were not significant (SIT: ES = −0.578 [−0.937, −0.220], *p* = 0.002 vs. HIIT: ES = −0.070 [−0.518, 0.377], *p* = 0.758). Both subgroups of HIIT and SIT resulted in lower AG concentration compared to MICT immediately post-exercise (SIT: −0.707 [−1.087, −0.327], *p* < 0.001, study arms = 7 vs. HIIT: ES = −0.775 [−1.318, −0.231], *p* = 0.005, study arms = 3). Visual inspection of the funnel plots showed evidence of publication bias in analysis of AG immediately and 30–90 min post-exercise. Egger's regression test was significant in the analysis of AG immediately post-exercise (intercept = −6.707, confidence interval: −9.43 to −3.98, *p* < 0.001), but was not significant in the analysis of AG at 30- to 90-min post-exercise.

**Fig. 2:**
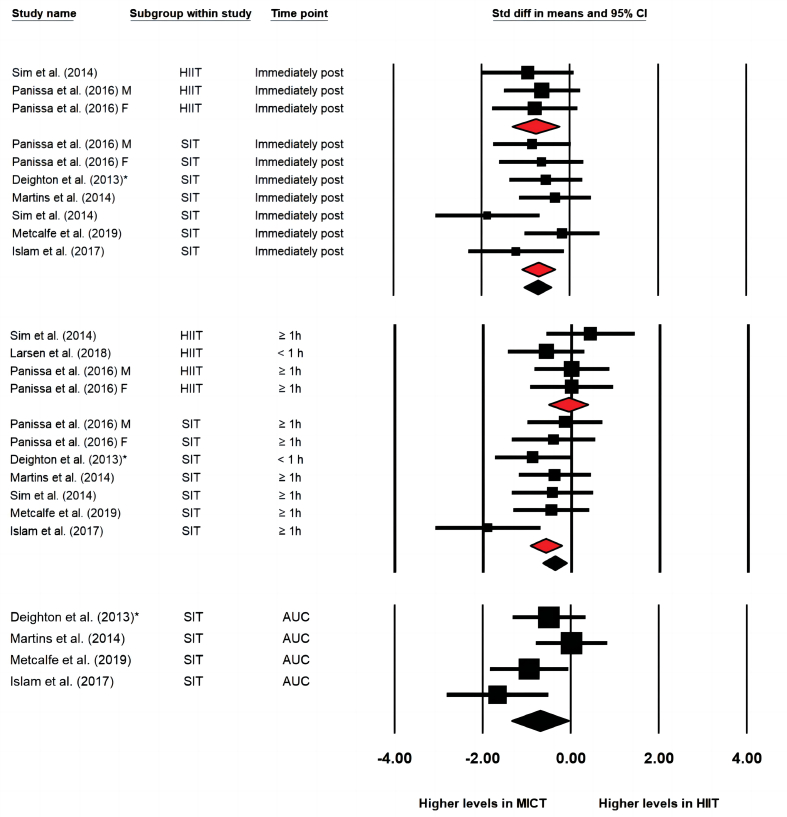
Forest plot of effects of HIIT/SIT in comparison to MICT on concentration of acylated ghrelin immediately post exercise, 30–90 min post exercise and the area under curve values (AUC); * = the latter published study that published in the same year by the same first author; F = group of females; M = group of males; CI = confidence interval.

#### 3.2.2 Total GLP-1

Five studies analysed total GLP-1 concentrations [22,25,27,[28], [37]] and four out of the five studies [25,27,[28], [37]] observed higher concentrations of total GLP-1 after HIIT/SIT and MICT compared to the control group. No differences between HIIT/SIT and MICT were found in three of the four studies (two studies analyzing active and normal-weight individuals [22,25]; one involving inactive and overweight subjects [27], while one study [28] observed greater increase in total GLP-1 after MICT compared to HIIT/SIT protocols with significantly lower work volume. Active GLP-1 concentration in active normal weight subjects after SIT was assessed in one study [26] and the result in this study showed increase in the exercise group compared to the control group. In this study, the concentration was greater in MICT immediately post-exercise but were greater in SIT 30-min post-exercise and in AUC values.

Outcomes of meta-analysis of the acute effect of the EXE compared to the CON on total GLP-1 concentrations were presented in supplementary Figure S3. Compared to the CON, exercises induced significantly higher total GLP-1 concentration immediately post-exercise (ES = 0.371 [0.013, 0.729], *p* = 0.042, study arms = 12, I^2^ = 50.074), 30- to 90-min post-exercise (ES = 0.905 [0.646, 1.165], *p* < 0.001, study arms = 12, I^2=^0) and in AUC values (ES = 0.483 [0.145, 0.821], *p* = 0.005, study arms = 8, I^2=^13.086). The effects sizes were large 30- to 90-min post-exercise but were small to moderate immediately post-exercise and in AUC values. Sensitivity analyses were performed with one study removed and the overall effects were not significantly driven by a single study. Visual inspection of the funnel plots and Egger's regression tests suggested potential publication bias in the analysis of GLP-1 immediately (intercept = 14.220, confidence interval: 7.21 to 21.10, *p* = 0.001) and 30–90 min post-exercise (intercept = 8.520, confidence interval: 3.29 to 13.75, *p* = 0.005). Results of subgroup and meta-regression analysis in the meta-analysis of GLP-1 immediately post-exercise (Table 2) demonstrated that gender elicited significant impact on the overall effect, while other moderators (i.e., BMI, fitness level and protocol type) did not significantly influence the effect size.

HIIT/SIT did not result in greater increases in total GLP-1 compared to MICT in the meta-analyses of effects immediately post-exercise (ES = −0.111 [−0.459, 0.238], *p* = 0.533 I^2=^0, study arms = 6), 30- to 90-min post-exercise (ES = 0.103 [−0.490, 0.695], *p* = 0.734, I^2^ = 63.6%, study arms = 6) or in AUC values (ES = −0.025 [−0.461, −0.411], *p* = 0.910, I^2^ = 0, study arms = 4) (Fig. 3). The one-study-removed procedure did not impact the overall effect. Subgroup analysis of differences between HIIT and SIT compared to MICT was not able to be performed due to insufficient numbers of study arms in the subgroup of HIIT. Visual inspection of funnel plot and Egger's test were not performed due to insufficient numbers of study arms (n < 10).

**Fig. 3:**
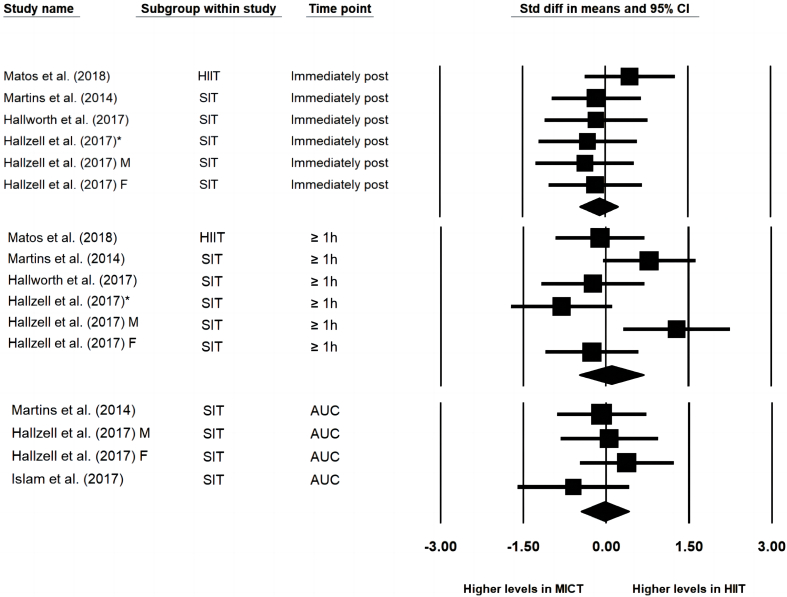
Forest plot of effects of HIIT/SIT in comparison to MICT on concentration of total GLP-1 immediately post exercise, 30–90 min post exercise and the area under curve values (AUC); * = the latter published study that published in the same year by the same first author; F = group of females; M = group of males; CI = confidence interval.

#### 3.2.3 Total PYY

Eight studies [16,[21], [22], [23], [25], [26],[35], [37]] assessed total PYY concentration after the exercises and half of the studies [16,23,25,35] did not find any changes in total PYY after HIIT/SIT and MICT compared to the non-exercise control group. Participants in one of the four studies did not directly report the fitness level of the subjects whose BMI was 25 ± 4 [16], while the other three studies involved either inactive and obese individuals (three studies) or lean and active people (one study). In the other half of the studies involving only active normal weight subjects that observed changes in total PYY concentration, results were contradictory between studies, with two favouring HIIT/SIT [[22], [37]], one favouring MICT [37] and one reporting no differences between HIIT/SIT and MICT [23].

Outcomes of meta-analysis of the acute effect of the EXE compared to the CON on total PYY concentrations were presented in supplementary Figure S4. There were significantly larger effects of the EXE compared to the CON on increasing total PYY concentration immediately post-exercise (ES = 0.892 [0.514, 1.270], *p* < 0.001, study arms = 17, I^2=^61.9%) and in AUC values (ES = 0.658 [0.224, 1.092], *p* = 0.003, I^2^ = 44.904, study arms = 8), but no greater effects were observed in the EXE 30- to 90-min post-exercise (ES = 0.210 [−0.012, 0.432], *p* = 0.064, I^2^ = 3.4%, study arms = 17). The one-study-removed procedure did not significantly change the overall effect. Visual inspection of the funnel plots suggested chances of publication bias in the analyses of PYY immediately and 30–90 min post-exercise. Egger's regression test (intercept = 9.980, confidence interval: 5.57 to 14.39, *p* < 0.001) was significant in the analysis of PYY immediately post-exercise but was nonsignificant in the analysis of PYY 30- to 90-min post-exercise. Subgroup and meta-regression analyses were performed in the meta-analysis of PYY immediately post-exercise (Table 2). The results indicated that fitness level (i.e., active), protocol type (i.e., MICT) and gender were associated with the overall effect.

HIIT/SIT protocols generated greater effects (i.e., moderate to large effects) than MICT immediately post-exercise (ES = 0.700 [0.361, 1.040], *p* < 0.001), study arms = 9, I^2^ = 15.9%), but resulted in similar effects compared with MICT 30- to 90-min post-exercise (ES = 0.032 [−0.248, 0.311], *p* = 0.825, study arms = 10, I^2^ = 0) and in AUC values (ES = 0.107 [−0.327, 0.541], *p* = 0.629, study arms = 4, I^2^ = 0) (Fig. 4). Subgroup analysis showed no significant differences between HIIT (ES = 0.162 [−0.478, 0.803], *p* = 0.619, study arms = 2) and MICT or SIT and MICT (ES = 0.001 [−0.310, 0.311], *p* = 0.996, study arms = 8) in the effects on PYY concentrations 30- to 90-min post-exercise. Sensitivity analysis did not show significant impact of single study on the overall effect. Subgroup analysis for effects immediately post-exercise and in AUC values were not performed due to insufficient numbers of study arms in the subgroup of HIIT. Potential publication bias was detected from the inspection of the funnel plot in the analysis of PYY 30- to 90-min post-exercise and the Egger's regression test was significant (intercept = −6.939, confidence interval: −12.35 to −1.53, *p* = 0.02).

**Fig. 4:**
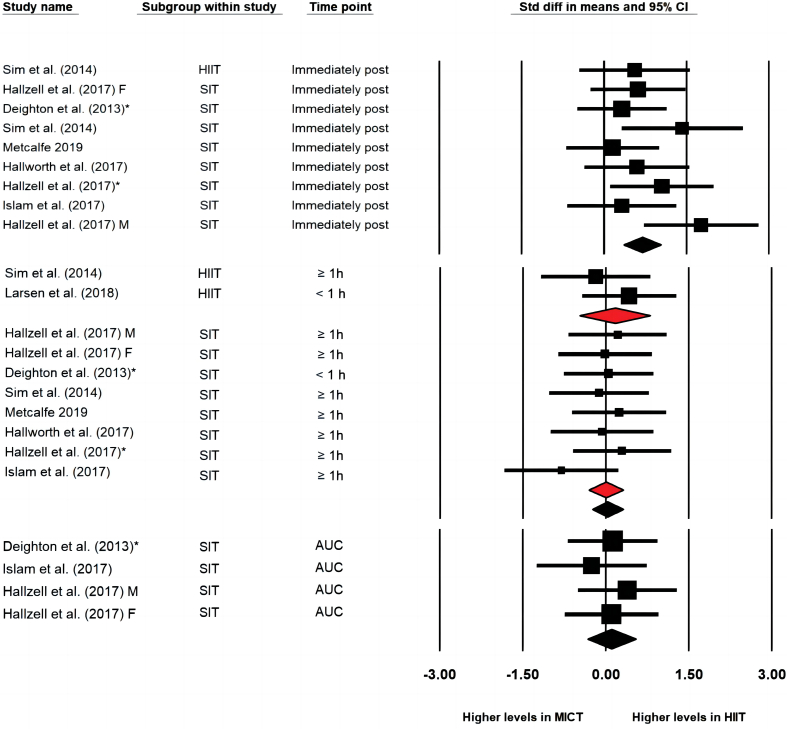
Forest plot of effects of HIIT/SIT in comparison to MICT on concentration of total PYY immediately post exercise, 30–90 min post exercise and the area under curve values (AUC); * = the latter published study that published in the same year by the same first author; F = group of females; M = group of males; CI = confidence interval.

#### 3.2.4 PYY_3-36_

Three studies [27,[24], [36]] reported data for the bioactive form of PYY and one of the studies involving normal weight individuals observed statistical changes in PYY_3-36_ after exercise compared to the control group; the results were favouring MICT immediately post-exercise but favored HIIT in the hours after exercise [24]. The other two studies which recruited either inactive overweight subjects [27] or active normal-weight subjects did not find any effects of exercise on PYY_3-36_ [36].

As shown in Figure S5, results of meta-analysis showed no statistically different effects in the comparisons of EXE to CON on PYY_3-36_ concentration at any of the analysed time points (immediately post: ES = 0.618 [−0.023, 1.258], *p* = 0.059, study arms = 10, I^2^ = 79.8%; 30- to 90- min post: ES = 0.151 [−0.422, 0.723], *p* = 0.606, study arms = 10, I^2^ = 75.9%) or in AUC values (ES = 0.180 [−0.221, 0.581], *p* = 0.379, study arms = 4, I^2^ = 0). The one-study-removed procedure did not significantly alter the overall effect. Visual inspection of funnel plots suggested evidence of publication bias in the analysis of PYY_3-36_ concentration immediately and 30- to 90- min post-exercise, but Egger's regression tests were not significant *(p* > 0.05).

HIIT/SIT did not generate greater effects compared to MICT immediately post-exercise (ES = −0.140 [−0.584, 0.303], *p* = 0.535, study arms = 6, I^2^ = 30.0%) or 30- to 90- min post-exercise (ES = −0.362 [−0.838, 0.115], *p* = 0.137, study arms = 6, I^2^ = 77.6%) (Fig. 5). The subgroup of SIT or the subgroup of HIIT did not result in significantly different effects compared to the MICT at the analysed time-points. Meta-analysis for AUC concentrations of PYY_3-36_ was not performed given the limited available data. Visual inspection of funnel plots and Egger's tests were not performed due to insufficient numbers of study arms (n < 10). The results of subgroup and meta-regression analysis in the meta-analysis of PYY_3-36_ immediately post-exercise (Table 2) demonstrated that fitness level (i.e., inactive) had a significant impact on the overall effect. However, it should be noted that no active participants were involved in the subgroup analysis of fitness level (i.e., study arms: inactive = 8, not reported = 2). Results of subgroup analysis and meta-regression of PYY_3-36_ 30- to 90-min post-exercise suggested that gender impacted the overall effects.

**Fig. 5:**
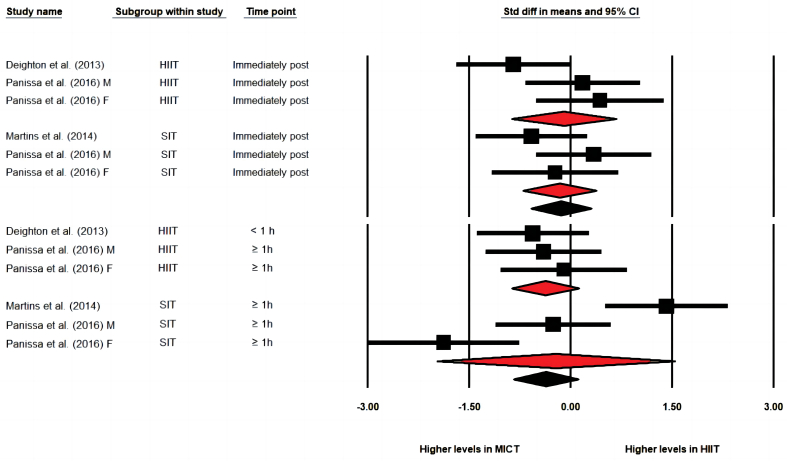
Forest plot of effects of HIIT in comparison to MICT on concentration of PYY3-36 immediately post exercise, 30–90 min post exercise; * = the latter published study that published in the same year by the same first author; F = group of females; M = group of males; CI = confidence interval.

## 4 Discussion

As the first systematic review and meta-analysis to compare the effects of HIIT/SIT and MICT on appetite-related hormones including AG, PYY, GLP-1 in healthy adults, the current study demonstrated that both HIIT/SIT and MICT could suppress plasma AG concentrations and increase total PYY and total GLP-1 concentrations compared to the non-exercise control group, while there were no significant effects of exercise on PYY_3-36_. In studies with direct comparisons between HIIT/SIT and MICT, greater suppression of AG (all analysed time points and AUC values) and an increase in total PYY (immediately post-exercise) were found in HIIT/SIT (i.e., SIT > HIIT) compared to MICT. However, contradictory to our hypothesis, no significant effects of exercise modality (HIIT/SIT vs. MICT) were found in total GLP-1 and PYY_3-36_ concentrations.

Acylated ghrelin is the bioactive form of ghrelin which is predominantly produced by endocrine cells in the stomach and acts as appetite stimulating signals to achieve energy homeostasis [8]. The results of the present study showed that both MICT and HIIT/SIT could induce a significant decrease in plasma AG concentrations compared to the control group, but HIIT/SIT protocols with higher intensity led to greater decreases in AG when directly compared to MICT protocols. Moreover, the outcomes of subgroup analysis suggested that the magnitude of the effects of interval trainings on decreasing AG concentrations to a greater extent than MICT was influenced by the intensity, as SIT generated greater effect sizes compared to HIIT with lower intensity. It seems that impact of the exercise volume is less powerful than intensity on suppressing AG production after exercise, since greater effects of HIIT/SIT were reported in both studies that matched work done in HIIT/SIT and MICT and studies that adopted HIIT/SIT protocols with a much lower exercise volume than MICT. One of the key mechanisms that has been proposed frequently to explain the impact of exercise intensity on circulating AG concentrations is blood redistribution [15]. During exercise, blood flow is redirected from splanchnic area to active skeletal muscle to meet the demand of physical movements, which reduced availability of oxygen in the stomach (i.e. exercise-induced hypoxia) [39] in a intensity-dependent manner, thus inhibiting the activation of ghrelin due to the decreased activity of the enzyme ghrelin O-acyltransferase [40] and slowing gastric emptying due to perturbed gastric motility [41]. This shared mechanism in humans might also help to explain the low heterogeneity levels and consistent outcomes regarding the superior effects of SIT followed by HIIT and MICT on AG suppression across studies involving participants with varied physiological characteristics (e.g., active or inactive).

In contrast to ghrelin, anorectic hormone GLP-1 is predominantly produced by L cells in small and large intestine in response to food intake and influence insulin and glucagon secretion to restrain hepatic glucose production [5]. Despite the fact that HIIT/SIT and MICT resulted in a significant increase in total GLP-1 concentration, the impact of intensity was not as distinct in GLP-1 responses to HIIT/SIT as in AG concentrations. It is possible that changes in GLP-1 concentrations after exercise could be more affected by training volume or energy expenditure rather than intensity, which is supported by some evidence from the included studies reporting greater effects of MICT with lower intensity but higher training volume than HIIT/SIT on increasing total GLP-1 [28] or active GLP-1 concentrations [26]. It is unknown based on the limited available data whether differences in participants characteristics, for example, body fat percentage, which has been suggested to attenuate post-prandial rise in GLP-1 [42], could affect GLP-1 responses to HIIT/SIT compared to MICT. In the analysis comparing exercise groups including HIIT/SIT and MICT with the CON, results of the meta-regression suggested that gender influenced the overall effect. However, it should be noted that there were uneven numbers of study arms in each subgroup with more studies in the subgroup of males (study arms = 13) than the subgroup of females (study arms = 4). In one study [37][22], [37] which directly compared the effects of SIT and MICT between males and females in GLP-1 responses, increased GLP-1 concentrations were found in females but not in males with lower body fat percentages immediately after exercise, which indicated the impact of sex differences in changes of GLP-1 after exercise.

PYY is another anorectic hormone that mainly secreted in intestinal tract proportionally in response to the calorie intake and especially fat intake [1]. In the current study, effects of exercise protocols including MICT and HIIT/SIT on increasing total PYY concentration were found in the meta-analysis of responses immediately post-exercise and in AUC values compared to the control group, but no significant effects were generated in the active form of PYY (PYY_3-36_) despite a small number of the included studies (n = 3) analysed PYY_3-36_. Although the effects of exercise on PYY concentrations were relatively small and transient, as presented in the current study, exercise intensity still exerted some influence on the effect sizes, as larger effects in HIIT/SIT compared to MICT were found immediately post-exercise. Nevertheless, the subgroup analysis comparing the effects of HIIT and SIT on PYY concentration was only able to be performed in meta-analysis for total PYY responses 30- to 90-min post-exercise due to limited data; no statistical differences were found between these two interval protocols, despite the studies in the HIIT subgroup (n = 2) being outnumbered by the studies in the SIT subgroup (n = 8). Therefore, more studies are needed to confirm the effects of higher intensity in HIIT/SIT compared to MICT on increasing total PYY or PYY_3-36_ concentrations. Moreover, although the fitness level and gender of the participants could also influence PYY responses, as seen in outcomes of the meta-regression analysis, further investigations are required to draw conclusions on the impact of these two moderators on PYY responses, as the majority of the studies involved males and active participants.

In the present study, the effect of exercise intensity on AG and PYY was pronounced immediately post-exercise and gradually weakened 30- to 90-min post-exercise, despite that the effect of HIIT/SIT on AG was still significantly different from that of the MICT 30- to 90-min post-exercise. In view of the significant greater attenuation of AG levels after HIIT/SIT compared to MICT, HIIT, especially SIT with reduced time commitment, may represent a more successful and effective approach in suppressing the hormone of hunger for individuals with needs to control appetite but report a lack of time. Given that the effects of SIT on suppression of AG could be achieved even with extremely low exercise volume, as demonstrated in one study analysing reduced-exertion HIIT (REHIT) with two sets of 20-s sprints in a 10-min low-intensity exercise session, the need to utilise exercise protocols with larger volume could be decreased when aiming to inhibit the generation of AG. Nevertheless, regarding the results that the higher intensity of HIIT/SIT did not elicit substantially different effects compared to MICT in PYY and GLP-1 concentrations, both MICT and HIIT could be beneficial for increasing the anorectic gastrointestinal hormones and potentially promoting satiety. Nevertheless, it should be acknowledged that the acute change in hormone levels after exercise is only one of the factors (e.g., post-exercise oxygen consumption and lactate level) [17,43,44] accounting for post-exercise energy intake and energy deficits created by performing the exercise. There are several limitations that should be mentioned when interpreting the findings of the present study. Firstly, as majority of the participants in the included studies were lean active males, the impact of individual differences, such as gender, body composition, fitness level, could not be clearly addressed in the current study. Although sub-group and meta-regression analyses were performed in analyses comparing exercise groups (HIIT/SIT and MICT) and the control groups, the results should be interpreted with caution, as the number of study arms in each subgroup were uneven in most of the analyses. Additionally, it has been suggested that exercise protocols causing larger mechanical disturbance (e.g. running) could induce greater effects on appetite-regulating hormones [14,45]. However, given that most studies in the current study utilised cycling as the exercise mode of HIIT/SIT and MICT, it remains to be investigated whether different exercise modes (running vs. cycling) could affect hormone responses to HIIT/SIT. Secondly, although the pooled effects of the meta-analysis were significant in the analysed hormones (e.g., AG, PYY), it should be kept in mind that hormone responses could be highly variable between individuals, as large standard deviations were reported in the majority of the included studies. Thirdly, although the present study conducted analyses using AUC values that were commonly used to demonstrate hormone concentrations by time [14], the amount of data was small and the calculations of AUC were variable in the studies included with different sampling rates, which might lower the precision of the meta-analyses. Nevertheless, we also analysed hormone responses immediately post-exercise and at 30- to 90-min post-exercise to capture acute changes in the hormones induced by HIIT/SIT and MICT across time. Lastly, as the impact of food intake could be substantial on appetite-regulating hormones [5], differences in the test-meals (i.e., breakfast) provided before exercise might affect hormone responses after exercise and confound the findings of the current study. In the present study, the majority of the included studies provided standardised breakfast before exercise consisting of 20–30% of the estimated daily energy intake with macronutrients of 10–15% proteins, 55–75% carbohydrates and 15–30% lipids, while two studies did not provide meals before exercise [16,23] and both studies reported suppressed AG but unaffected total PYY concentrations after HIIT/SIT and MICT.

In conclusion, the present study provided valuable information on the effects of HIIT/SIT compared to MICT on appetite regulating hormones. Both HIIT/SIT and MICT suppressed AG and increased GLP-1 and PYY concentrations but did not induce significant effects on PYY_3-36_. Importantly, in an intensity-dependent manner, interval protocols resulted in greater effects in reducing AG levels compared to MICT, while the impact of exercise intensity on GLP-1 and PYY was less strong. Overall, SIT protocols with lower volume but higher intensities could be more favourable exercise options for reducing AG levels and suppressing hunger compared to MICT.

## Author contribution statement

All authors listed have significantly contributed to the development and the writing of this article.

## Funding statement

Dr Zhaowei Kong was supported by 10.13039/501100004733University of Macau [MYRG2020-00266-FED].

## Data availability statement

Data associated with this study has been deposited at “Systematic Review Registration” under the accession number [CRD42022337521] (https://www.crd.york.ac.uk/PROSPERO).

## Additional information

Supplementary content related to this article has been published online at [URL].

## Declaration of interest's statement

The authors declare no conflict of interest.

## Figures and Tables

**figs1:**
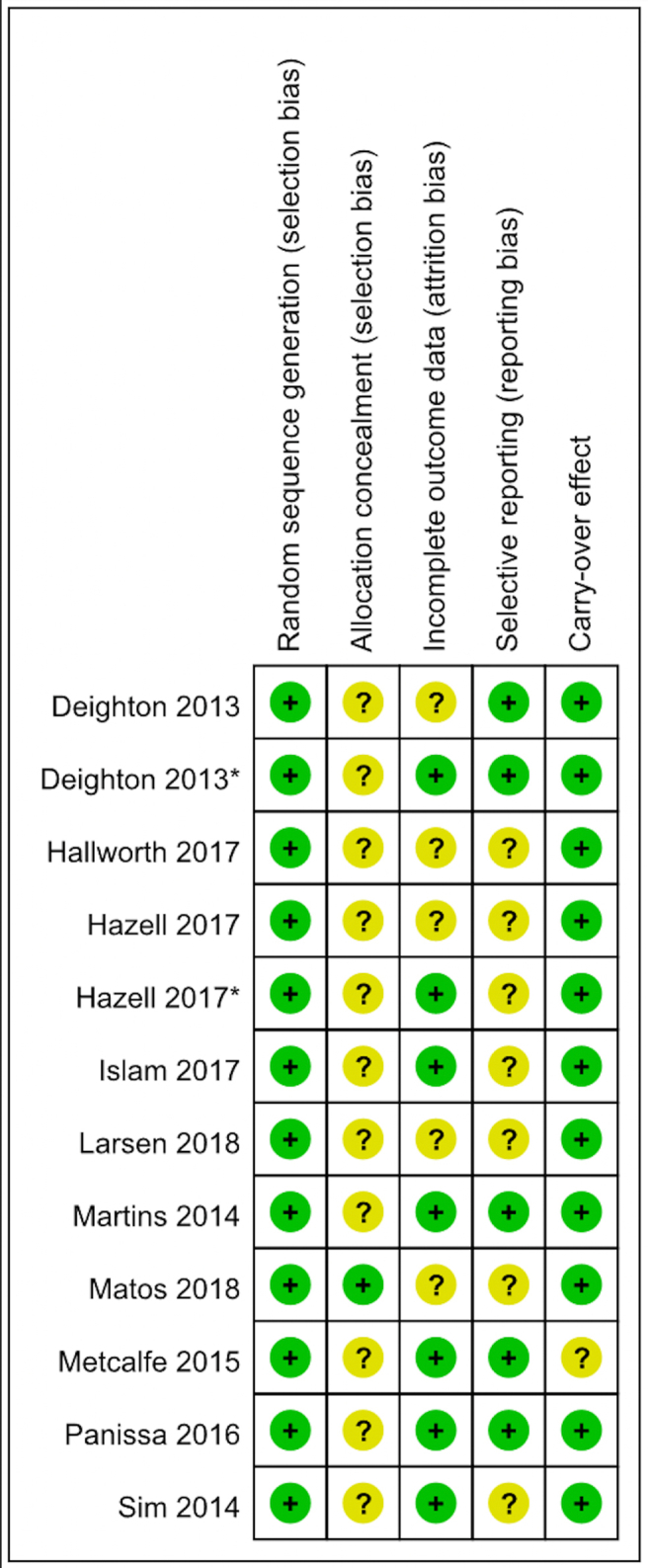


**figs2:**
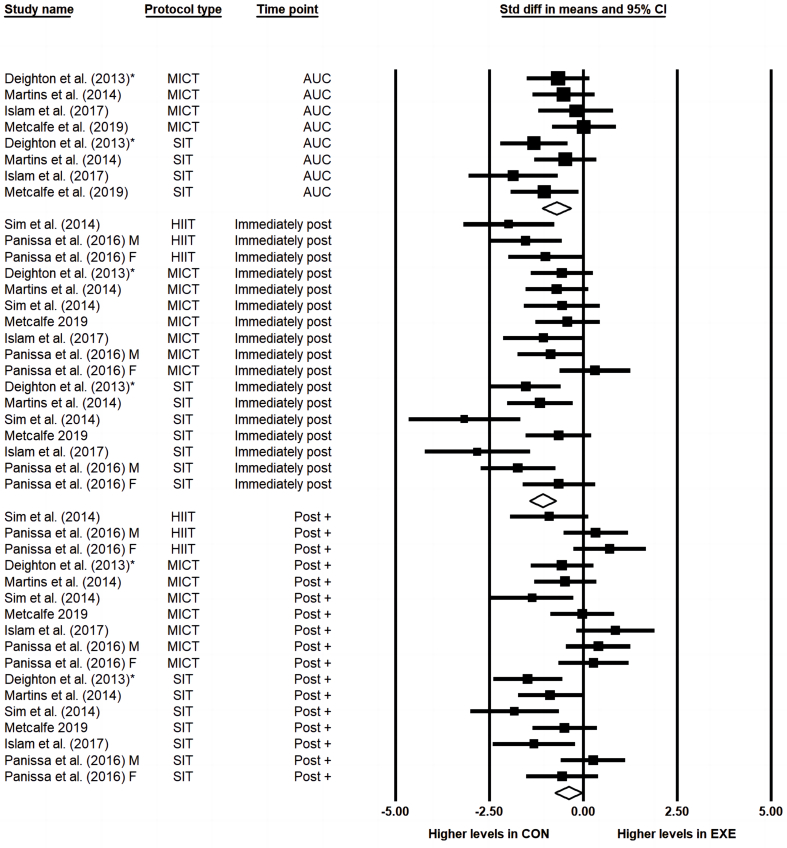


**figs3:**
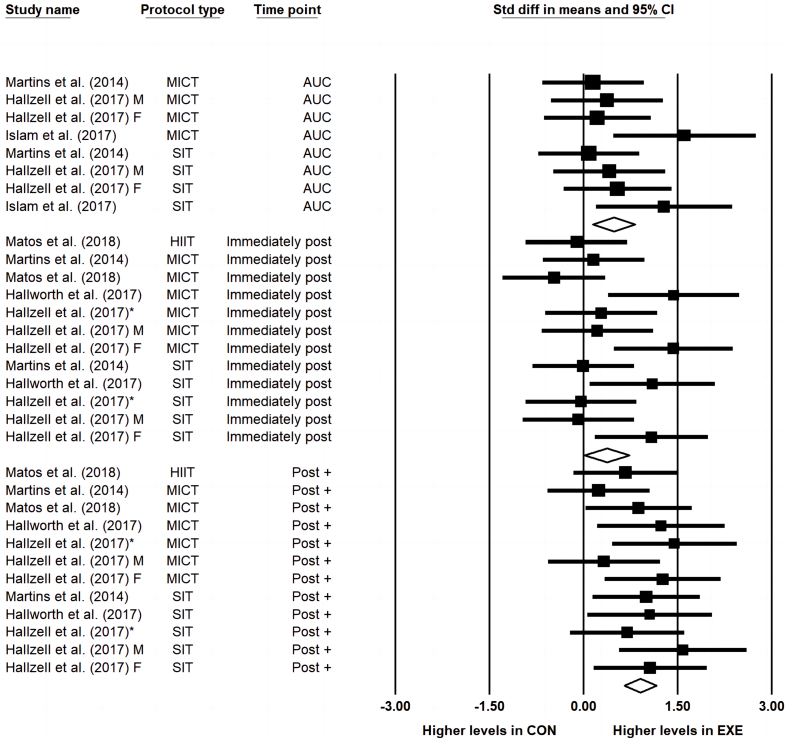


**figs4:**
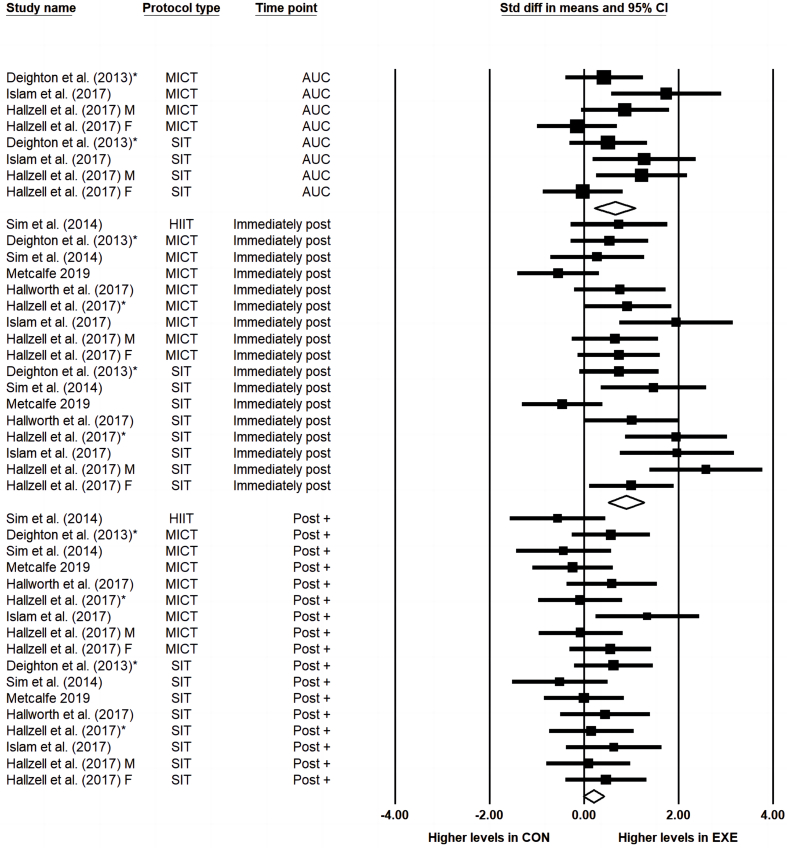


**figs5:**
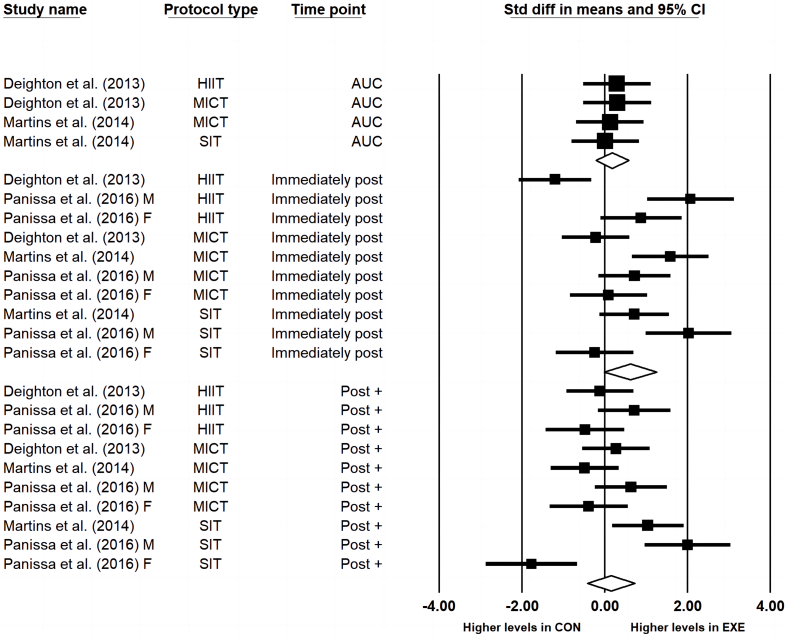


**figs6:**
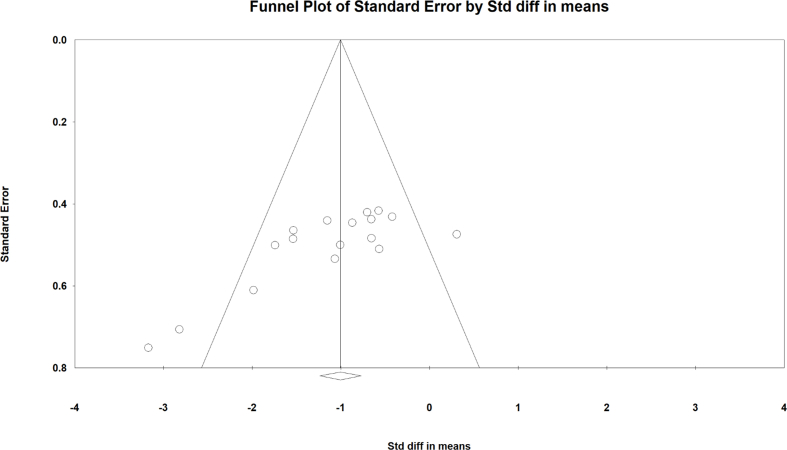


**figs7:**
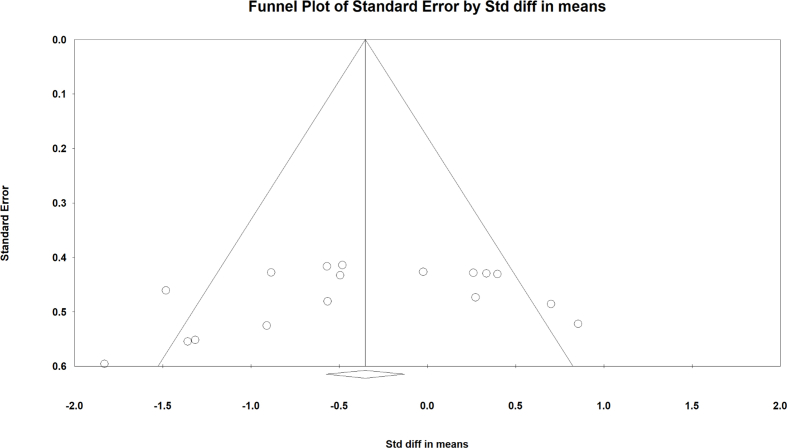


**figs8:**
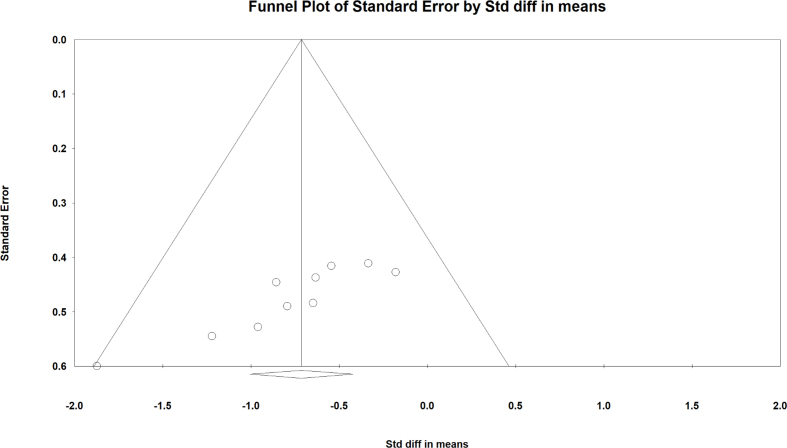


**figs9:**
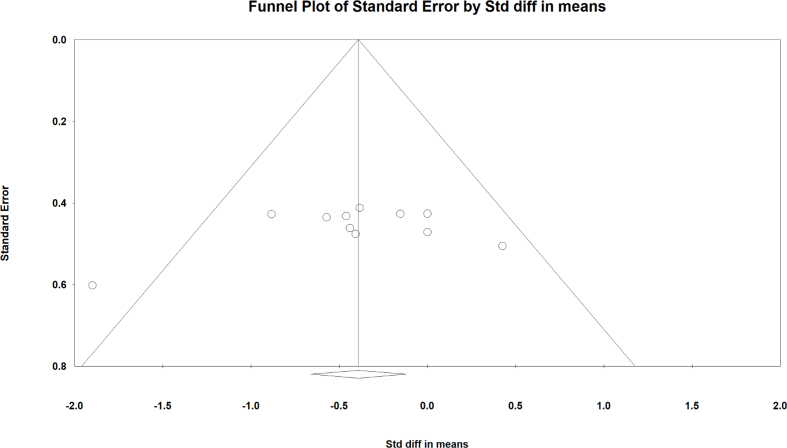


**figs10:**
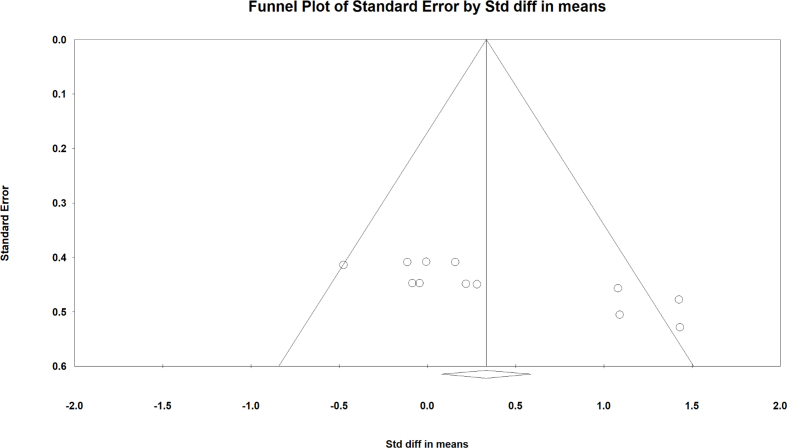


**figs11:**
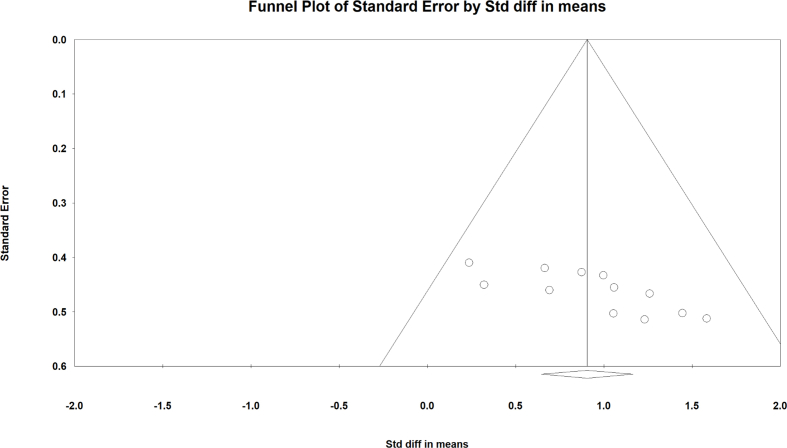


**figs12:**
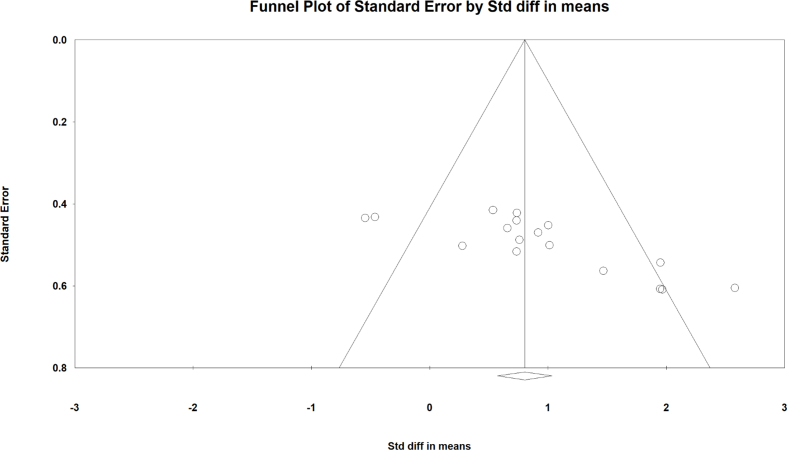


**figs13:**
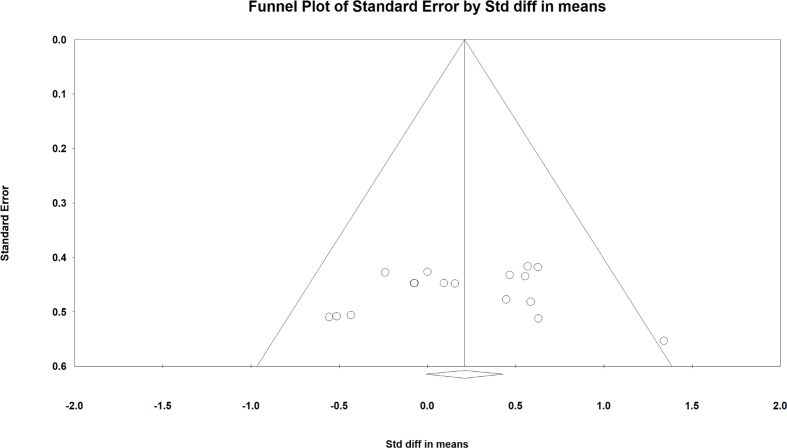


**figs14:**
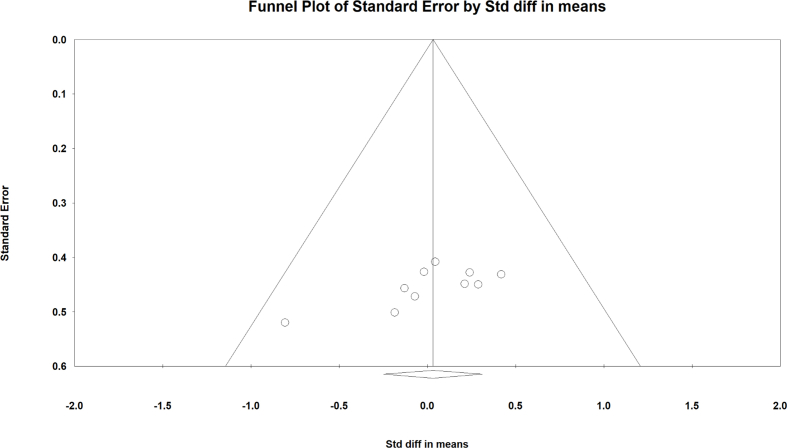


**figs15:**
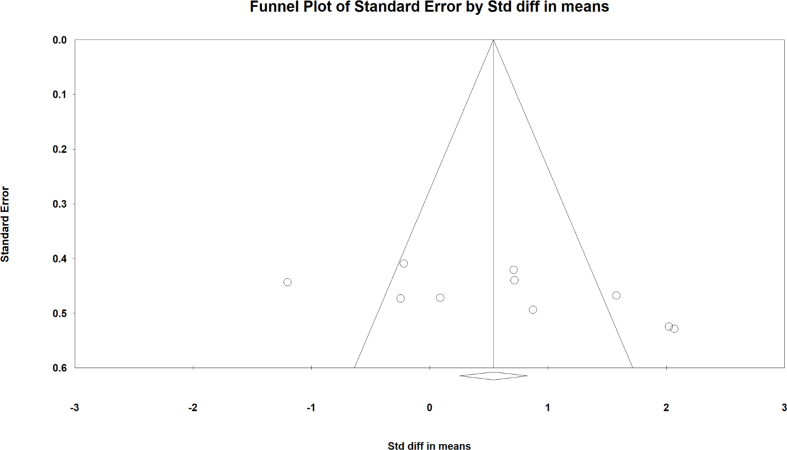


**figs16:**
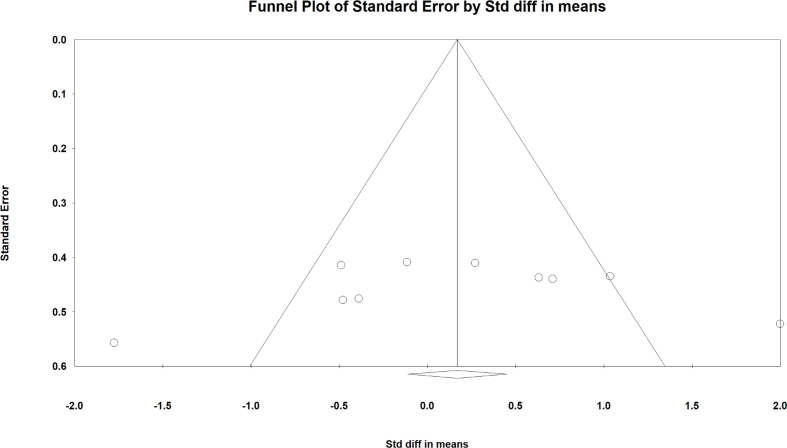

