# Supplementary material for: Acute effect of high-intensity interval training versus moderate-intensity continuous training on appetite-regulating gut hormones in healthy adults: A systematic review and meta-analysis
Source: Heliyon. 2023 Jan 21;9(2):e13129. doi: 10.1016/j.heliyon.2023.e13129 (PMC9898666; doi:10.1016/j.heliyon.2023.e13129)
Supplement: Table_S1 [file mmc1.docx]

**Table S1** Search strategy and key words

| **Intervention** | **Outcome** |
| --- | --- |
| 1. exercise | 4. appetite |
| 1. physical activity | 5. appetite hormone |
| 1. high intensity training | 6. gastrointestinal hormone |
|  | 7. appetite-regulating hormone |
|  | 8. ghrelin |
|  | 9. acylated ghrelin |
|  | 10. PYY |
|  | 11. peptide YY |
|  | 12. PYY3-36 |
|  | 13. glucagon-like peptide-1 |
|  | 14. active GLP-1 |
|  | 15. GLP-1(7-36) |
|  | 16. GLP-1(9-36) |
| **Search strategy:** | |
| 1 and 4 or 5 or 6 or 7 or 8 or 9 or 10 or 11 or 12 or 13 or 14 or 15 or 16 | |
| 2 and 4 or 5 or 6 or 7 or 8 or 9 or 10 or 11 or 12 or 13 or 14 or 15 or 16 | |
| 3 and 4 or 5 or 6 or 7 or 8 or 9 or 10 or 11 or 12 or 13 or 14 or 15 or 16 | |
