# Supplementary material for: Acute effect of high-intensity interval training versus moderate-intensity continuous training on appetite-regulating gut hormones in healthy adults: A systematic review and meta-analysis
Source: Heliyon. 2023 Jan 21;9(2):e13129. doi: 10.1016/j.heliyon.2023.e13129 (PMC9898666; doi:10.1016/j.heliyon.2023.e13129)
Supplement: Table_S2 [file mmc2.docx]

**Table S2** Summary for the outcomes of meta-analysis

| **Outcome** | **Comparisons** | | **Time point** | | |
| --- | --- | --- | --- | --- | --- |
|  |  |  | **Immediately post exercise** | **30 – 90 min post exercise** | **AUC** |
| **Acylated ghrelin** | HIIT/SIT/MICT (**EXE**) **vs.** control (**CON**) | | ES = -1.078 [-1.427, -0.729], p < 0.001, study arms = 17, I^2^=54.8% | ES = -0.387 [-0.747, -0.026], p = 0.035, study arms =17, I^2^=61.8% | ES = -0.705 [-1.090, -0.320], p < 0.001, study arms =8, I^2^=33.4% |
|  | **HIIT/SIT vs. MICT** | HIIT and SIT: | ES = -0.729 [-1.040, -0.418], p < 0.001, study arms =10*, I^2^ = 0% | ES = -0.380 [-0.659, -0.100], p = 0.008, study arms=11*, I^2^ = 18.3% | ES = -0.696 [-1.345, -0.047], p = 0.036, study arms =4, I^2^ = 52.5%^#^ |
|  |  | Subgroup of HIIT: | ES = -0.775 [-1.318, -0.231], p = 0.005, study arms = 3 | ES = -0.070 [-0.518, 0.377], p = 0.758 study arms = 4 | N/A, study arms = 0 |
|  |  | Subgroup of SIT: | ES =-0.707 [-1.087, -0.327],  p < 0.001, study arms = 7 | ES = -0.578 [-0.937, -0.220], p = 0.002, study arms = 7 | N/A |
| **Total**  **GLP-1** | HIIT/SIT/MICT (**EXE**) **vs.** control (**CON**) | | ES = 0.371 [0.013, 0.729], p =0.042, study arms =12, I^2^=50.1% | ES = 0.905 [0.646, 1.165], p < 0.001, study arms =12, I^2^ = 0% | ES = 0.483 [0.145, 0.821], p =0.005,  study arms =8, I^2^ = 13.1% |
|  | **HIIT/SIT vs. MICT** | HIIT and SIT: | ES = -0.111 [-0.459, 0.238], p =0.533  study arms =6, I^2^ = 0% | ES = 0.103 [-0.490, 0.695], p =0.734,  study arms = 6, I ^2^= 63.6%^#^ | ES = -0.025 [-0.461, -0.411], p =0.910,  study arms = 4, I^2^=0% |
|  |  | Subgroup of HIIT: | N/A, study arms = 1 | N/A, study arms = 1 | N/A, study arms = 0 |
|  |  | Subgroup of SIT: | N/A | N/A | N/A |
| **Total**  **PYY** | HIIT/SIT/MICT (**EXE**) **vs.** control (**CON**) | | ES= 0.892 [0.514, 1.270], p < 0.001, study arms = 17, I^2^ = 61.9% | ES = 0.210 [-0.012, 0.432], p = 0.064,  study arms = 17, I^2^ = 3.4% | ES= 0.658 [0.224, 1.092], p =0.003  study arms = 8, I^2^=44.9% |
|  | **HIIT/SIT vs. MICT** | HIIT and SIT: | ES= 0.700 [0.361, 1.040], p < 0.001), study arms = 9, I^2^=15.9% | ES= 0.032 [-0.248, 0.311], p =0.825,  study arms = 10*, I^2^=0% | ES=0.107 [-0.327, 0.541], p =0.629  study arms = 4, I^2^=0% |
|  |  | Subgroup of HIIT: | N/A, study arms = 1 | ES= 0.162 [-0.478, 0.803], p =0.619, study arms = 2 | N/A, study arms = 0 |
|  |  | Subgroup of SIT: | N/A | ES= 0.001 [-0.310, 0.311], p =0.996, study arms = 8 | N/A |
| **PYY_3-36_** | HIIT/SIT/MICT (**EXE**) **vs.** control (**CON**) | | ES= 0.618 [-0.023, 1.258], p =0.059, study arms = 10, I^2^=79.8% | ES= 0.151 [-0.422, 0.723], p = 0.606, study arms = 10, I^2^=75.9% | ES= 0.180 [-0.221, 0.581], p =0.379  study arms = 4, I^2^=0% |
|  | **HIIT/SIT vs. MICT** | HIIT and SIT: | ES= -0.140 [-0.584, 0.303], p =0.535  study arms = 6, I^2^=30.0% | ES= -0.362 [-0.838, 0.115], p = 0.137, study arms = 6, I^2^=77.6%^#^ | N/A, study arms = 0 |
|  |  | Subgroup of HIIT: | ES= -0.098 [-0.869, 0.674], p = 0.80, study arms = 3 | ES= -0.373 [-0.869, 0.122], p = 0.140,  study arms = 3 | N/A |
|  |  | Subgroup of SIT: | ES= -0.161 [-0.703, 0.381], p =0.560, study arms = 3 | ES= -0.216 [-1.973, 1.542], p = 0.810  study arms = 3 | N/A |

ES= effect size; [ , ] = 95% confidence interval; N/A = data not available; * = study arms≥10; ^#^ = moderate to high heterogeneity in analysis of HIIT/SIT vs. MICT
